# Reduction in incomplete stent apposition area caused by jailed struts after single stenting at left main bifurcation lesions: micro-CT analysis using a three-dimensional elastic bifurcated coronary artery model

**DOI:** 10.1007/s12928-016-0380-6

**Published:** 2016-03-11

**Authors:** Yutaka Hikichi, Mitsuo Umezu, Koichi Node, Kiyotaka Iwasaki

**Affiliations:** 1 Cooperative Major in Advanced Biomedical Sciences, Joint Graduate School of Tokyo Women’s Medical University and Waseda University, Waseda University, 2-2 Wakamatsucho, Shinjuku, Tokyo Japan; 2 Department of Cardiovascular Medicine, Saga University, Saga, Japan; 3 Faculty of Science and Engineering, Waseda University, Shinjuku, Japan

**Keywords:** Left main bifurcation, Incomplete stent apposition, Jailed strut, Stent expansion method, Stent design

## Abstract

Stent struts protruding into ostial side branch called “jailed strut” at bifurcation lesions is a likely cause of thrombus formation. We aimed to investigate the influences of multiple kissing balloon inflation (KBI) for stent expansion, and stent platform design, respectively, on the reduction of incomplete stent apposition area (ISA area) caused by jailed struts at a side-branch ostium, using a three-dimensional elastic left main (LM) bifurcated coronary artery model. The referenced LM bifurcation angle data of 209 patients were stratified by tertiles focusing on the angle between the LM trunk (LMT) and left anterior descending artery (LAD). A bifurcation model was fabricated with angles of 129°, 122.2°, and 76.4° for LMT–LAD, LMT–left circumflex (LCx), and LAD–LCx, respectively, and with diameters of 5, 3.75, and 3.5 mm for LMT, LAD, and LCx, respectively; these diameters fulfill Murray’s law. A 75 % stenosis was included along the LMT. One-time and three-time KBIs were conducted using two-link Nobori and three-link Xience Xpedition (*n* = 6 each). The ISA area was quantified using micro-CT. Three-time KBI was effective in reducing the ISA area compared with one-time KBI for both the Nobori (*p* = 0.05) and Xience Xpedition (*p* = 0.07). The ISA area was smaller in the Nobori than in the Xience Xpedition, both in one-time and three-time KBI (one-time KBI: *p* = 0.003; three-time KBI: *p* = 0.001). Our findings of this study on reducing the ISA area by focusing on an interventional technique and stent design may help to improve coronary bifurcation intervention for a possibly better long-term clinical outcome.

## Introduction

Drug eluting stents (DESs) have been shown to reduce the rate of restenosis considerably, and have widely expanded treatments for various types of severe lesions and indications of percutaneous coronary intervention (PCI) [1, 2]. However, coronary bifurcation lesions, which account for 15–20 % of all PCIs, still represent a challenging lesion subset for interventional cardiologists [3, 4]. Several studies have reported that a two-stent (complex stent) strategy does not offer any advantage over a single-stent (simple stent) strategy in bifurcation lesions [5, 6]. Therefore, single-stent implantation might be the first line strategy in bifurcation lesions. Although coronary artery bypass graft surgery (CABG) has been considered the standard method for treating unprotected left main coronary artery (ULMCA) bifurcation disease according to the current guidelines [7, 8], DESs have been used with increasing frequency for the PCI of ULMCA bifurcation diseases [9, 10]. A recent intravascular ultrasound (IVUS) study indicated that the presence of incomplete stent apposition (ISA), confirmed 8 months after DES implantation, was associated with a higher rate of myocardial infarction and very late stent thrombosis (VLST) at a 5-year follow-up. [11]. An optical coherence tomography (OCT) study of bifurcation lesions treated with a single DES showed that the frequency of thrombus attachment at side-branch orifices was lower with kissing balloon inflation (KBI) as compared to the frequency of thrombus attachment without KBI, possibly because of the reduction in jailed struts [12]. The Korean multicenter registry trial demonstrated that the single DES strategy with KBI was associated with a lower rate of major adverse cardiac events (MACE) and target lesion revascularization (TLR) than the single DES strategy without KBI [13]. Some reports suggested the importance of KBI from the in vitro and in vivo findings of the optimization of bifurcation treatments [14, 15]. However, little is known about the effects of the stent expansion method and stent platform design on the clinical outcome. We hypothesized that stent thrombosis after single DES stenting may occur even after KBI owing to residual stent struts protruding into the ostial side branch called “jailed strut” in bifurcation lesions. Here, we investigated the influences of stent implantation methods and stent platform design on the ISA area caused by jailed struts at the side-branch ostium, using a bifurcated elastic coronary artery model to gain insights into more sophisticated single stenting strategies.

## Methods

### Left main bifurcation model

**Fig. 1:**
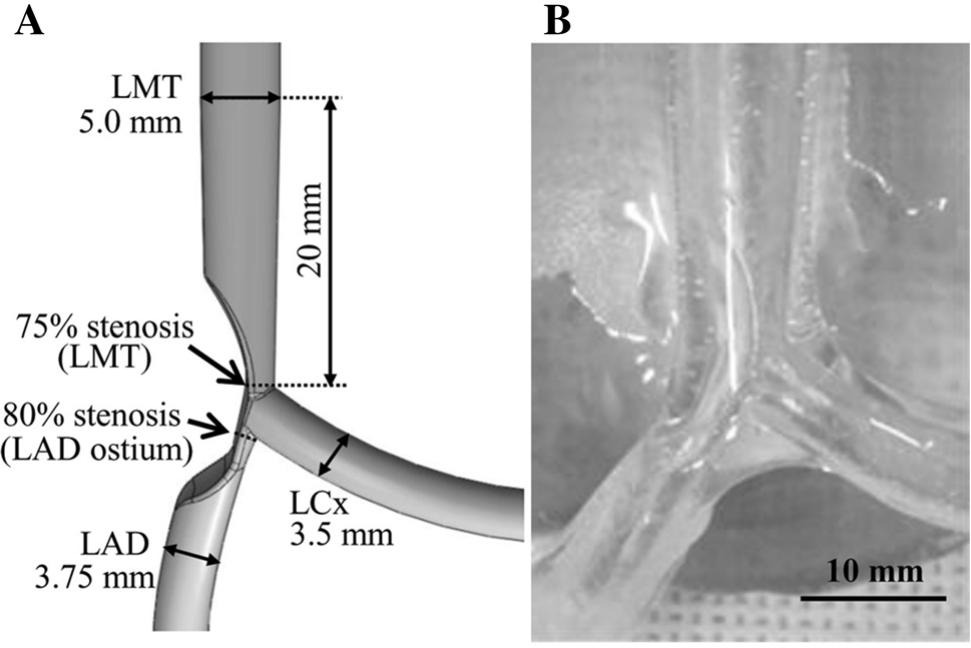
Three-dimensional elastic left main bifurcated coronary artery model. **a** The dimensions of the left main bifurcation model. **b** The elastic left main bifurcation models employed in the bench test

Left main (LM) bifurcation angle data from 209 de novo patients who underwent 64 multi-slice computed tomography, reported by Kawasaki et al. [16], were used to construct the LM coronary artery bifurcation model. The angles between the left main trunk (LMT) and the left anterior descending artery (LAD) were focused on in this study, and the angle data from the 209 patients were stratified by tertiles. Based on each mean value, the narrow-angled three-dimensional LM bifurcation model with LMT–LAD, LMT–left circumflex (LCx), and LAD–LCx angles of 129.0°, 122.2°, and 76.4°, respectively, was fabricated (Fig. 1a, b). The LM bifurcation model had 75 % stenosis along the LMT, and 80 % stenosis at the LAD ostium. The model was constructed with reference vessel diameters of 5.0 mm for LMT, 3.75 mm for LAD, and 3.5 mm for LCx. These diameters followed Murray’s law [17]. The tract from the LMT to the two distal bifurcated arteries was curved by 120°.

### Effect of stent expansion times on ISA caused by jailed struts

We speculated that repeating KBI after single stenting might reduce ISA caused by jailed struts at the LCx ostium. In this study, the effect of three-time KBI on ISA was compared with that of one-time KBI (*n* = 6 each).

### Effect of stent platform design on ISA caused by jailed struts

We speculated that stent platform design might influence the ISA caused by jailed struts at the LCx ostium. In this study, the two-link stainless steel Nobori stent (diameter and length of 3.5 and 24 mm, respectively; Terumo, Tokyo, Japan) and three-link cobalt chromium Xience Xpedition (Xience) stent (diameter and length of 3.5 and 23 mm, respectively; Abbott Vascular, Illinois, USA) were used to investigate the effect of stent platform design on the ISA caused by jailed struts at the LCx ostium (*n* = 6 each). The 3.5-mm-diameter Nobori stent with 10 crowns and two links is only available in Japan; the 3.5-mm-diameter Nobori stent with 9 crowns and three links is available in the US and EU.

### Stent deployment procedure

**Fig. 2:**
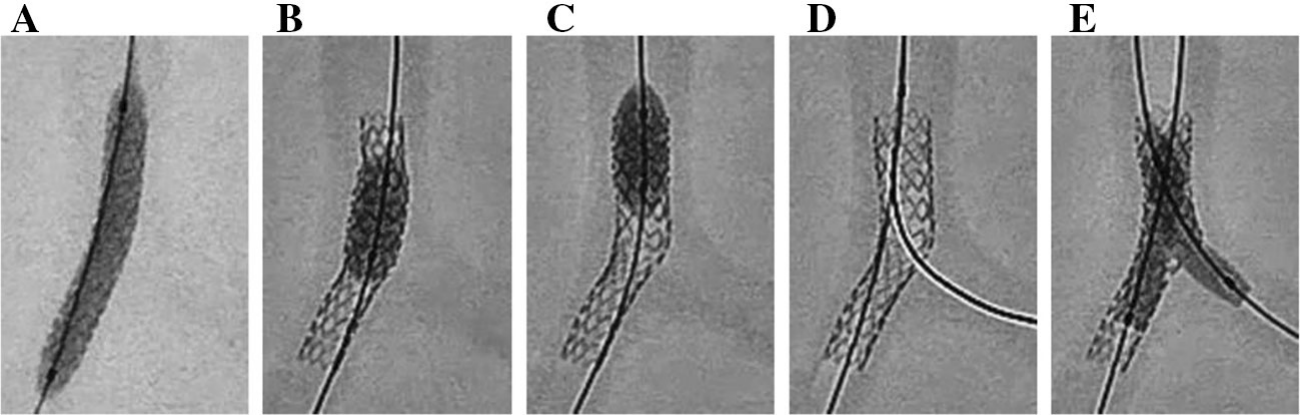
Stent deployment procedures. **a** Stent deployment along LMT–LAD. **b**, **c** Proximal optimization technique. **d** Advancement of guidewire through a distal stent cell accessing the LCx. **e** Kissing balloon inflation

All procedures were conducted under X-rays under the same conditions as those employed in clinical practice (Fig. 2). The Nobori stent and Xience stent were deployed along LMT–LAD with balloon inflation pressures of 14 and 16 atm, respectively (Fig. 2a). The stent delivery balloon was inflated three times, each time for 20 s. Then, the proximal optimization technique was conducted using a balloon with a diameter and length of 5.0 mm and 8 mm (Fig. 2b, c). A guidewire was then advanced through a distal stent cell accessing the LCx (Fig. 2d). Then, KBI was conducted one time and three times, 20 s each, for the two stents (Fig. 2e). Semi-compliant balloons with diameters and lengths of 3.5 and 15 mm, respectively, were used, and expanded at the balloon inflation pressures based on the compliance chart in accordance with 3.75-mm-diameter LAD and 3.5-mm-diameter LCx ostium.

### Quantification of ISA caused by jailed struts

**Fig. 3:**
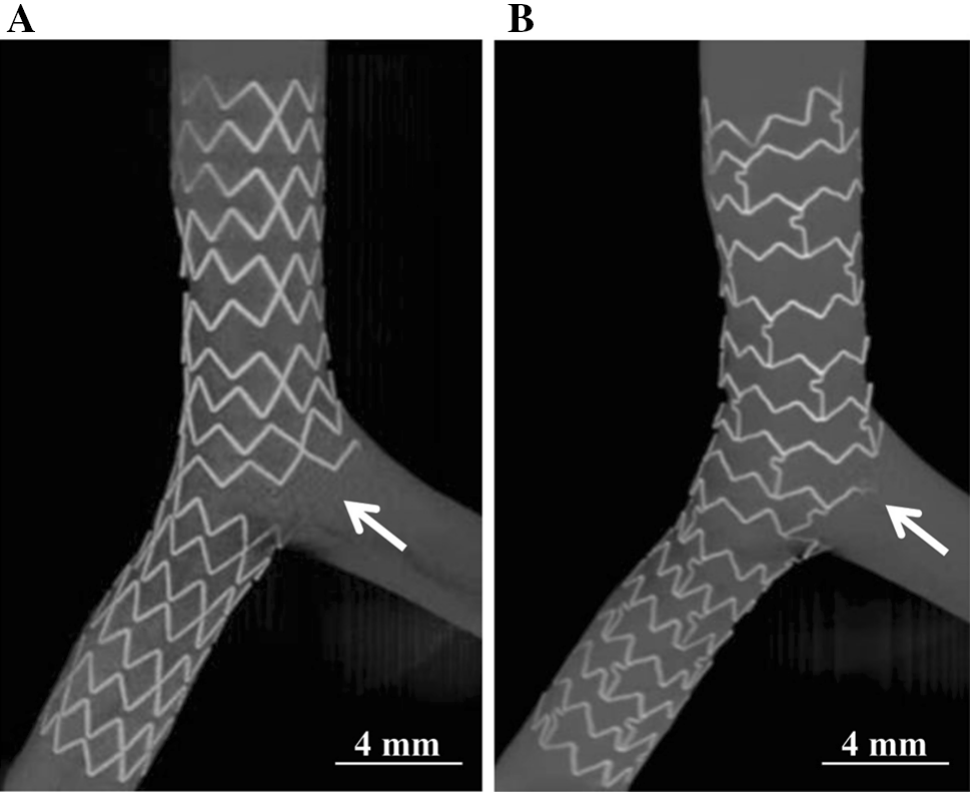
Micro-CT analysis of the incomplete stent apposition (ISA) area caused by jailed struts at the LCx ostium. **a** The two-link Nobori stent deployed in the bifurcation model. **b** The three-link Xience Xpedition stent deployed in the bifurcation model

The ISA caused by jailed struts was quantified using a micro-CT (TDM 1300-IS, Yamato Scientific Co., Ltd., Japan). The lumen of the bifurcated models was filled with a 70 wt% radiopaque contrast medium (Baritop Sol 150, Sakai Chemical Industry Co., Ltd., Japan) for the micro-CT analysis. The three-dimensional structure of the stents was reconstructed using 512 CT slices with a spatial resolution of 0.048 mm (Fig. 3a, b). The ISA area was defined as the area between jailed stent struts and the vessel wall of the LCx ostium. The ISA area was then measured at the cross section in perpendicular with the LCx flow tract based on the LMT-LCx angle.

### Statistical analysis

Continuous data are presented as the medium and quarter tile. For primary analysis of the ISA area caused by jailed struts, the continuous data were compared using Levene’s test. Comparisons of continuous data with not-normal distribution were conducted using the Welch test. The student-*t* test was used to compare continuous data with normal distribution. The SPSS software version 21 (IBM Corporation, Armonk, New York) was used for these analyses. A value of *p* < 0.05 was considered statistically significant.

## Results

### One-time KBI versus three-time KBI

**Fig. 4:**
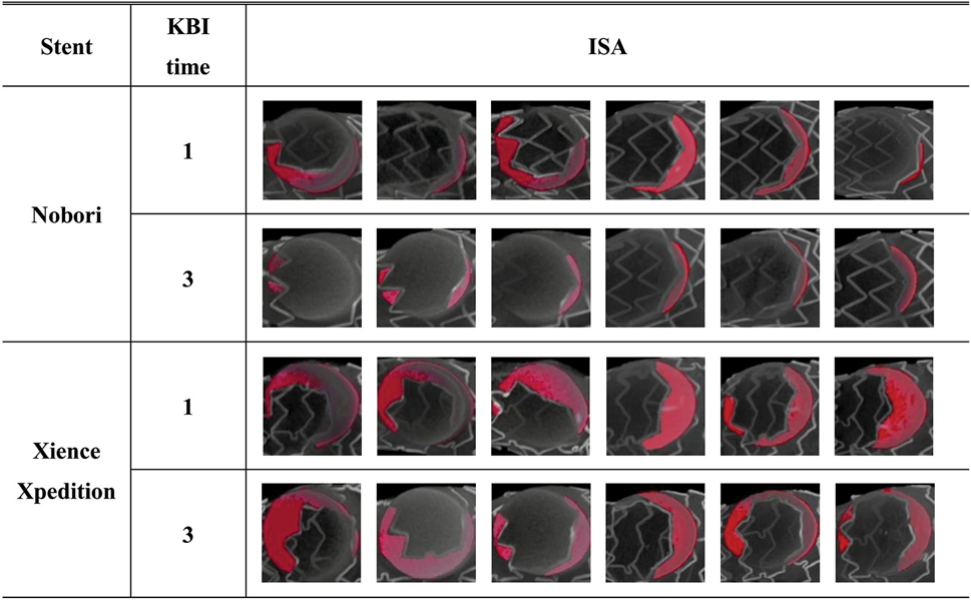
Incomplete stent apposition areas detected by the micro-CT analysis. For each condition, six stents were tested

**Fig. 5:**
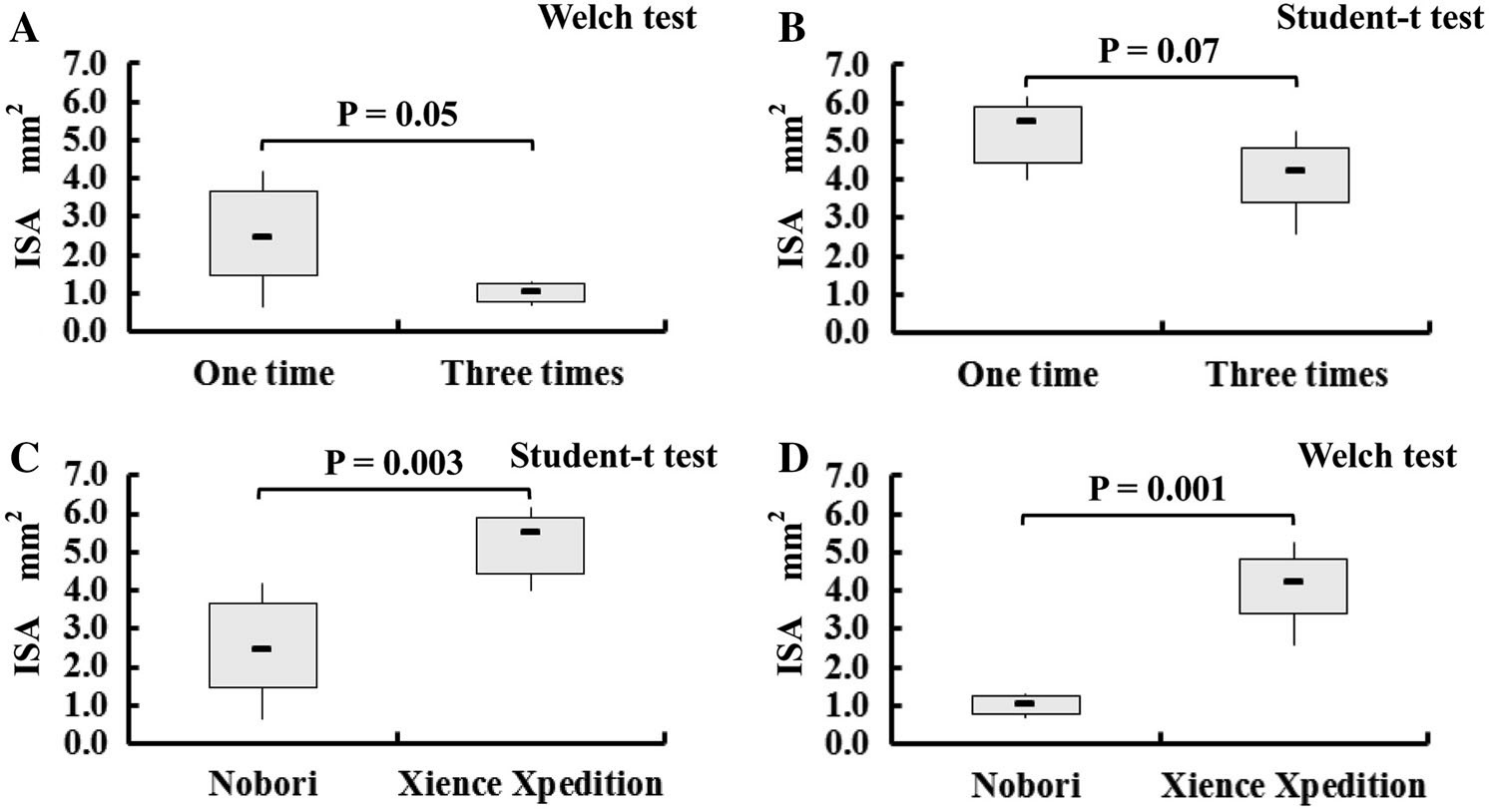
Effects of multiple kissing balloon inflation (KBI) and stent platform design on the incomplete stent apposition (ISA) area caused by jailed struts at the LCx ostium. **a** Comparison between one-time KBI and three-time KBI using the two-link Nobori stent. **b** Comparison between one-time KBI and three-time KBI using the three-link Xience Xpedition stent. **c** Comparison between the two-link Nobori stent and the three-link Xience Xpedition stent with one-time KBI. **d** Comparison between the two-link Nobori stent and the three-link Xience Xpedition stent with three-time KBI

The ISA areas detected by the micro-CT analysis were summarized in Fig. 4. The ISA area caused by jailed struts at the LCx ostium was lower with the three-time KBI than the ISA area caused by jailed struts at the LCx ostium with the one-time KBI for the Nobori stent (Nobori: 1.00 ± 0.28 mm^2^ vs. 2.49 ± 1.44 mm^2^, *p* = 0.05; Xience: 4. 07 ± 1.04 mm^2^ vs. 5.21 ± 0.93 mm^2^, *p* = 0.07) (Fig. 5a, b). For the Nobori stents, the variance in the ISA caused by jailed struts became distinctly smaller with three-time KBI compared to the variance with one-time KBI. As for the Xience stents, the three-time KBI had showed a positive effect with regard to reducing the ISA compared with the one-time KBI.

### Impact of stent platform design

The ISA area caused by jailed struts at the LCx ostium using the Nobori stent was distinctly lower than that using the Xience stent, both with one-time KBI and three-time KBI (one-time KBI: 2.49 ± 1.44 mm^2^ vs. 5.21 ± 0.93 mm^2^, *p* = 0.003; three-time KBI: 1.00 ± 0.28 mm^2^ vs. 4. 07 ± 1.04 mm^2^, *p* = 0.001) (Fig. 5c, d).

## Discussion

The main findings of this study are as follows.

(1) The three-time KBI in single stenting was effective in reducing the ISA area caused by jailed struts at the LCx ostium, compared with the one-time KBI, for the two-link Nobori stent. (2) The two-link Nobori stent exhibited a distinctly lower ISA area caused by jailed struts at the LCx ostium than the three-link Xience stent, both with three-time KBI and one-time KBI.

Bifurcation treatment is recognized as a strong predictor of stent thrombosis [18]. ISA was defined as one or more struts clearly separated from the vessel wall with evidence of blood speckles behind the strut [19]. The ISA is recognized to pose certain risks for LST and VLST [20–23]. Recent IVUS and OCT studies in patients with LST or VLST confirmed that the ISA is associated with LST and VLST [21, 23, 24]. Hariki et al. reported that the KBI significantly reduced the jailed struts at the side-branch ostium and the frequency of thrombus attachment, possibly because of the reduction in the number of jailed struts [12]. In this study, we demonstrated that three-time KBI with single stenting in LMT to the LAD at LM bifurcation was effective in reducing ISA area caused by jailed struts at the LCx ostium. Moreover, the two-link Nobori considerably reduced the ISA compared with the three-link Xience stent irrespective of one-time KBI or three-time KBI, indicating the importance of selecting the stent platform at LM bifurcations.

## Limitations

The bifurcation model remains motionless during the stenting procedure, whereas the in vivo coronary artery moves owing to cardiac contraction and relaxation. Therefore, data deviations resulting from the produce in this study may be underestimated compared with in vivo situations. Nevertheless, a sophisticated bench test system would help to gain an understanding of better stent implantation strategies at bifurcation lesions.

## Conclusion

This bench study using a three-dimensional elastic bifurcated coronary artery model revealed that three-time KBI in single stenting is effective in reducing the ISA area caused by jailed struts at the LCx ostium in comparison with the general one-time KBI. Moreover, the choice of stent platform with a larger expansion capacity of stent cell has a considerable effect on the reduction of the ISA both with one-time KBI and three-time KBI. Our findings may help to improve the coronary bifurcation intervention for better clinical outcomes.
